# Impact of Implementing a Dyslipidemia Management Guideline on Cholesterol Control for Secondary Prevention of Ischemic Heart Disease in Primary Care

**DOI:** 10.3390/ijerph17228590

**Published:** 2020-11-19

**Authors:** Emma Forcadell Drago, Maria Rosa Dalmau Llorca, Carina Aguilar Martín, Ignacio Ferreira-González, Zojaina Hernández Rojas, Alessandra Queiroga Gonçalves, Carlos López-Pablo

**Affiliations:** 1 Equip d’Atenció Primària Tortosa Oest, Institut Català de la Salut, 43500 Tortosa, Tarragona, Spain; eforcadellg.ebre.ics@gencat.cat; 2 Programa de Doctorat en Medicina, Universitat Autònoma de Barcelona, 08035 Barcelona, Spain; nachoferreira@secardiologia.es; 3 Unitat de Suport a la Recerca Terres de l’Ebre, Fundació Institut Universitari per a la Recerca a l’Atenció Primària de Salut Jordi Gol i Gurina (IDIAPJGol), 43500 Tortosa, Tarragona, Spain; zojahernandez@gmail.com (Z.H.R.); aqueiroga@idiapjgol.info (A.Q.G.); 4 GAVINA Research Group, 43500 Tortosa, Tarragona, Spain; 5 Equip d’Atenció Primària Tortosa Est, Institut Català de la Salut, 43500 Tortosa, Tarragona, Spain; 6 Programa de Doctorat de Biomedicina, Universitat Rovira i Virgili, 43201 Reus, Spain; 7 Unitat d’Avaluació, Direcció d’Atenció Primària Terres de l’Ebre, Institut Català de la Salut, 43500 Tortosa, Tarragona, Spain; 8 Departament de Cardiologia, Vall d’Hebron Institut de Recerca (VHIR), Hospital Universitari Vall d’Hebron, 08035 Barcelona, Spain; 9 Centro de Investigación Biomédica en Red de Epidemiología y Salud Pública (CIBERESP), 28029 Madrid, Spain; 10 Unitat Docent de Medicina de Família i Comunitària Tortosa-Terres de L’Ebre, Institut Català de la Salut, 43500 Tortosa, Tarragona, Spain; 11 Departament de Patologia, Hospital de Tortosa Verge de la Cinta, Institut Català de la Salut, 43500 Tortosa, Tarragona, Spain; clopezp.ebre.ics@gencat.cat; 12 Institut d’Investigació Sanitària Pere Virgili (IISPV), 43500 Tortosa, Tarragona, Spain; 13 Departament d’Infermeria, Campus Terres de l’Ebre, Universitat Rovira i Virgili, 43500 Tortosa, Tarragona, Spain

**Keywords:** practice guideline, cholesterol, secondary prevention, ischemic heart disease, primary health care

## Abstract

Cardiovascular diseases (CVD) are the main cause of death worldwide. The control of CVD risk factors, such as dyslipidemia, reduces their mortality rate. Nonetheless, fewer than 50% of patients with ischemic heart disease (IHD) have good cholesterol control. Our objective is to assess whether the level of participation of general practitioners (GPs) in activities to implement a dyslipidemia management guideline, and the characteristics of the patient and physician are associated with cholesterol control in IHD patients. We undertook a quasi-experimental, uncontrolled, before-and-after study of 1151 patients. The intervention was carried out during 2010 and 2011, and consisted of a face-to-face training and online course phase (Phase 1), and another of face-to-face feedback (Phase 2). The main outcome variable was the low-density lipoprotein cholesterol (LDL-C) control, whereby values of <100 mg/dL (2.6 mmol/L) were set as a good level of control, according to the recommendations of the guidelines in force in 2009. After Phase 1, 6.7% more patients demonstrated good cholesterol control. With respect to patient characteristics, being female and being older were found to be risk factors of poor control. Being diabetic and having suffered a stroke were protective factors. Of the GPs’ characteristics, being tutor in a teaching center for GP residents and having completed the online course were found to be protective factors. We concluded that cholesterol control in IHD patients was influenced by the type of training activity undertook by physicians during the implementation of the GPC, and patient and physician characteristics. We highlight that if we apply the recent targets of the European guideline, which establish a lower level of LDL-C control, the percentage of good control could be worse than the observed in this study.

## 1. Introduction

Cardiovascular disease (CVD) is the leading cause of death worldwide. According to the World Health Organization, 16,295,000 people died from CVD in 2016, representing 29% of all deaths. Within the world ranking of the 20 main causes of death, ischemic heart disease (IHD) ranks first, accounting for 17% of all deaths [1]. In addition, CVD is associated with high morbidity rates and, especially because it affects a wide range of ages, its health, economic and social consequences are very important [2].

More than 50% of the reduction in CVD mortality is related to the amelioration of the most important cardiovascular risk factors (CVRFs), dyslipidemia being one of these [3,4]. Statins are a group of lipid-lowering drugs that reduce low-density lipoprotein cholesterol (LDL-C) levels and thereby cardiovascular morbidity and mortality, and the need for coronary interventions [3,5]. For this reason, statins are recommended for all patients with IHD [3,5,6,7]. However, in practice, fewer than 50% of IHD patients present good cholesterol control [3,8,9].

In order to overcome this situation, clinical practice guidelines (CPGs) have been developed that should improve the quality of care and management of these diseases [10]. However, the CPGs need to be strategically implemented to be able to overcome the obstacles in the environment in which they will be used, to improve adherence and, consequently, boost the health of the population [11].

Several studies have focused on evaluating different CPG implementation strategies. The use of educational material has been found to have a modest effect on their implementation, clinical sessions have a small influence, and passive dissemination is generally ineffective. However, audit and feedback are potentially effective strategies for influencing the success of implementation [12]. The advent of the Internet has enabled online learning, which, compared with face-to-face programs, offers the advantages of distance learning, flexibility and adaptability [13]. Internet-based learning is about as effective as traditional methods [14], although interactivity, practical exercises and repetition of concepts may encourage better learning results [15].

Regarding the implementation of CPGs for dyslipidemia, at the primary care level, changes in behavior and knowledge have been observed in online learning groups, which have led to an increase in the percentage of patients with high cardiovascular risk who were treated appropriately [16]. On the other hand, the combination of face-to-face sessions and online material had a modest effect on the management and prescription variables and no clear effects on the control of LDL-C [17].

A recent study conducted in 18 European countries (including Spain) found that fewer than a fifth of very high-risk cardiovascular patients accomplished their control cholesterol targets as recommended under current guidelines, because only a fraction of them take the adequate medication. This highlights the continuing need to implement the CPGs adequately [18]. CPGs must be implemented successfully to ensure good adherence to them, although studies are needed to establish the most effective strategies for achieving this.

A collaboration between primary and hospital care units in our region was initiated in 2008 to coordinate, harmonize and improve the treatment of patients with CVD. Among the activities, a multidisciplinary CPG has been developed for treating dyslipidemia as part of secondary prevention of CVD. The CPG implementation among professionals involved the combined use of several of the methodologies known to be the most effective (auditing, feedback and interactive online training) to see whether this strategy could help improve control of LDL-C in patients with IHD.

The aim of this study was to assess whether the level of participation by general practitioners (GPs) in activities related to the implementation of the CPG, together with the characteristics of patients and physicians, are associated with the control of LDL-C of patients with IHD.

## 2. Material and Methods

### 2.1. Study Design and Participants

A quasi-experimental, uncontrolled, before-and-after study was carried out on IHD patients attended by primary care GPs from the 11 primary care teams (PCTs) of the Gerència Territorial Terres de l’Ebre from Institut Català de la Salut (ICS).

### 2.2. Sample Size and Patient Selection

Accepting an alpha risk of 0.05 and a beta risk of less than 0.2, assuming that the initial percentage in LDL-C control is 50% and the minimum detectable difference is 7%, and performing a bilateral contrast, the minimum sample size is 892 patients. A loss-to-follow-up rate of 10% has been assumed.

The study included patients diagnosed and registered in the primary care electronic medical record (EMR) with a diagnosis of IHD. Patients were excluded if they: did not meet the criteria of the population served (minimum of 3 primary care visits in the previous 2 years, and at least 1 visit in the previous year); presented the coronary event during the 6 months before the start of the study (marginal time for the LDL-C to improve after the diagnosis of IHD); had a terminal illness; had not had their LDL-C levels measured in 2009 or 2011; changed physicians during the CPG implementation process.

### 2.3. Data Collection

Initially, a database was created containing the cases diagnosed with IHD, linked to their primary care physician (GP) in order to be able to relate the control of each patient’s LDL-C to the characteristics of their physician. The information about patient characteristics was mostly extracted from the computerized registry program used in primary care known as ECAP (Estació Clínica d’Atenció Primària), which was designed by the ICS. The ECAP is an EMR that incorporates diagnosis, clinical variables, prescription data and laboratory test results [19]. Since 2006, ICS professionals have received, every month, a report on their performance through the ECAP. Performance is calculated from relevant evidence-based clinical indicators, which are called EQA (Estàndard de Qualitat Assistencial) and the report contains feedback and a list of patients with a clinical situation that can be improved [20,21].

The information of the physicians was obtained from registry of the Information Technology (IT) Department. Information about attendance at the sessions and the online course was obtained through the training unit’s signed attendance sheets and Moodle platform, respectively, and was entered into the study database manually by a single person. Finally, an identity code was assigned to each record to enable the individuals to be anonymized.

### 2.4. Variables

Data were collected about the percentage of patients achieving good LDL-C control after the intervention, the percentage and level of participation of physicians in implementation activities, and the characteristics of the patients (age, sex, cardiovascular comorbidity, diabetes, hypertension, stroke, peripheral arthropathy, atrial fibrillation, smoking habit) and the physicians (sex, age, type of contract, belonging to a teaching center for GP residents (TCGPR), level of participation in the implementation). The main outcome variable was the control of LDL-C in the IHD patients, whereby values of <100 mg/dL (2.6 mmol/L) indicate a good level of control, according to the recommendations of the guidelines in force in 2009 [22,23,24].

### 2.5. Intervention

The CPG included recommendations for the control of CVRFs and a description of dyslipidemia treatment in patients with CVD, and was based on National Cholesterol Education Program (NCEP), American Heart Association/American College of Cardiology (AHA/ACC) and European Society of Cardiology (ESC) guidelines [22,23,24]. The recommended treatments were: non-pharmacological (diet and physical exercise) and pharmacological (statins: simvastatin, pravastatin, atorvastatin and rosuvastatin; fibrates: gemfibrozil, bezafibrate and fenofibrate; resins: cholestyramine; ezetimibe and omega 3 acids). Additionally, an algorithm for action was proposed for improving the control and monitoring of these patients. The CPG was published in the intranet of ICS.

The intervention involved several face-to-face training activities, self-audit, feedback and interactive online training, which was aimed at primary care GPs for the purpose of implementing the CPG.

The intervention was organized in two phases:

(a) Phase 1: face-to-face training session and online course

During February and March 2010, a face-to-face training session was held in each of the 11 PCTs, led by two physicians. Sessions were programed within the training schedule during normal working hours and lasted for 2 h. The aim of the sessions was to disseminate the CPG content, imparting a short version of the guideline, explaining the different secondary prevention measures of CVD and how to achieve appropriate levels of LDL-C. In addition, the research project was explained to the physicians and they were also encouraged to participate in the online course.

The 20-h interactive online course was available on the Moodle platform of the ICS from March to June 2010. The platform enabled course participants to have access to the relevant reading material, to carry out and submit activities, and to communicate with teachers. The teachers were physicians from the Institute of Pharmacology of the Autonomous Community of Catalonia. The dissemination of the course was performed through the intranet and by sending three e-mail reminders to each professional. The objectives of the online course were to make physicians aware of the existence of the new CPG, for them to understand how to apply its recommendations, and to improve the control of dyslipidemia in secondary prevention. The distinguishing feature of the course was that each physician should perform a self-audit that consisted of a review of their IHD patients who had poor LDL-C control, through consultation of the EQA alert in the ECAP, with the aim of proposing improvements in their degree of control.

(b) Phase 2: face-to-face feedback session

This phase was carried out during 2011 in each of the 11 PCTs. The session lasted 1 h and was performed by the same two physicians who led the face-to-face training session. The participants were reminded of the key points of the guideline. A group feedback session was held in which the overall results of the difference in LDL-C levels before and after the intervention were presented and discussed, taking into account the extent of participation in Phase 1.

### 2.6. Statistical Analysis

After debugging the database, the Kolmogorov-Smirnov test was used to determine whether continuous variables were normally distributed. Descriptive summaries were produced: frequencies and percentages for categorical variables; means and standard deviations for normally distributed continuous variables; medians and interquartile ranges for non-normally distributed continuous variables.

The proportions of the different groups or categories of the qualitative variables were examined using the Chi square test. McNemar’s test was used to compare values of qualitative variables of matched pairs of patients before and after the intervention. For continuous variables, differences between two groups, or more than two groups were assessed using Student’s t test or one-way ANOVAs (normally distributed variables), or the Mann-Whitney U or Kruskal-Wallis test (non-normally distributed variables), respectively.

We used multilevel logistic regression to evaluate the association of the different variables with LDL-C control, with patients as the first level and physicians as the second level. The degree of association of variables was expressed using odds ratios (ORs) and their 95% confidence intervals (CIs).

To estimate the proportion of the variance explained by the physicians’ characteristics, the variance partition coefficient (VPC) was calculated for the multilevel logistic regression using the formula of Snijders-Bosko [25].

In order to determine how much a patient’s risk would change from being attended by the physician with the highest risk of poor control to being attended by the physician with the lowest risk, we calculated the median odds ratio (MOR) [26].

All statistical analyses were performed using IBM SPSS Statistics v.21.0 (IBM, Armonk, NY, USA) and Stata Statistical Software v.15.0 (StataCorp, College Station,, TX, USA). The significance level was set at 5%.

### 2.7. Ethics

The project was approved by the Ethics Committee of the Fundació Institut Universitari per la recerca a l’Atenció Primària de Salut Jordi Gol i Gurina (IDIAPJGol) in Barcelona on 12 June 2009, with reference number P09/25.

## 3. Results

The sample consisted of 1151 patients diagnosed with IHD who met the inclusion criteria. Their median age was 72.3 years (standard deviation (SD) = 10.4 years), they exhibited a median of two associated cardiovascular comorbidities, and the majority were men (63.2%). A total of 19.2% of patients were assigned to PCTs linked to the TCGPR.

The sample included 108 primary care GPs. The majority were men (57.3%), the median age was 49.5 years (SD = 9.1 years), and 19 worked in a PCT linked to the TCGPR.

In Phase 1, 56.5% of the GPs, who were caring for 651 patients, attended the face-to-face training session. The online course was taken by 43.3% of GPs, who were responsible for 498 patients.

To analyze the results with respect to the level of participation in Phase 1, we classified the patient sample into four groups: those whose physicians had (a) not participated in any implementation activity, (b) only attended the face-to-face training session, (c) only participated in the online course, and (d) undertaken both activities.

Table 1 shows the distribution of participation of the physicians in the various activities of Phases 1 and 2 of implementation, and the number of patients involved. In Phase 1, 67.6% of the physicians participated in at least one of the activities offered. In Phase 2, fewer than half of the physicians attended the feedback session.

### 3.1. Changes Observed in LDL-C Control after Implementing the CPG

To evaluate Phase 1, the 2011 LDL-C levels of 1151 patients were analyzed. For Phase 2, the 2012 data of 912 patients were evaluated. Both phases were analyzed one year after the completion of their corresponding activities of CPG implementation.

Table 2 shows that, of the patients who had good control of LDL-C before Phase 1, 11.3% exhibited poor control after the implementation of Phase 1, and 18.0% who initially had poor control achieved good control. Thus, after Phase 1, 6.7% more patients exhibited good control of LDL-C than before (*p* < 0.001).

Table 2 also illustrates that, of the patients with good control of LDL-C before Phase 2, 19.1% exhibited poor control afterwards, and that 26.4% of those with poor control after Phase 2 achieved good control. In short, after Phase 2, there were 7.3% more patients with good LDL-C control than before. However, the differences were not statistically significant (*p* = 0.128).

### 3.2. Features Associated with LDL-C Control after Phase 1

Regarding the characteristics of the patients that could be associated with poor LDL-C control, being female and being older were identified as risk factors. Otherwise, being diabetic was a protective factor. Of the physicians’ characteristics, being a tutor in a TCGPR was a protective factor for patients against poor control of LDL-C (Table 3).

To assess whether being a tutor in a TCGPR was a protective factor against patients’ poor LDL-C control based on the intensity of participation in Phase 1, a new composite variable was created (“level of participation by TCGPR tutoring status” in Table 4). Table 4 shows that being a tutor in a TCGPR and having completed only the online course was associated with a higher percentage of patients who controlled their LDL-C.

The proportion of the variance explained (PVE) indicates that 21.46% of the variability of LDL-C control after Phase 1 may be explained by the characteristics of the patient and the physician. The variance partition coefficient (VPC) indicates that physicians account for 3% of the variability in the LDL-C control. Considering the physician as the second level, the median odds ratio (MOR) per physician was 1.33, meaning that there was a 33% difference in the risk of poor LDL-C control between the physician with the highest risk and the physician with the lowest risk of poor LDL-C control.

### 3.3. Factors Associated with the Control of LDL-C after Phase 2

Table 5 shows that, of the patient characteristics, suffering a stroke was a protective factor against poor control of LDL-C, whereas none of physician variables was associated with LDL-C control.

The PVE indicates that 16.92% of the variability of LDL-C control after Phase 2 may be explained by the characteristics included in the model, and the VPC indicates that physicians account for 3% of the variability. The MOR per physician of 1.36 means that there is a 36% difference in the risk of poor LDL-C control between the physician with the highest risk and the physician with the lowest risk of poor LDL-CD control.

## 4. Discussion

Following the preparation of a CPG for managing dyslipidemia as part of secondary prevention of CVD, an implementation strategy was developed, consisting of face-to-face training, an interactive online course, self-audit and feedback session with the aim of evaluating its impact on the control of LDL-C in IHD patients.

Participation by GPs was around 50% for the face-to-face sessions, and 40% for the online course. 32.4% of the physicians did not participate in any proposed activity, although the face-to-face training was scheduled in the training calendar of the PCT during normal working hours.

In our study, which was conducted under the real conditions of daily clinical practice, the attendance rate was lower than in previous studies developed as clinical trials. In these trials, 66.7–80.0% and 69.6–86.0% of physicians in control and intervention groups, respectively, attended CPG presentation sessions [17,27], whereas in our study 67.6% and 46.3% participated in Phases 1 and 2, respectively.

The implementation of Phase 1 (the face-to-face training session and the online course), resulted in 6.7% more patients having good LDL-C control, since more patients improved their lipid control than those whose control became worse. In Phase 2 (the feedback session), although the percentage of patients with good control increased, the change was not statistically significant.

The intervention consisted of training physicians to improve their patients’ control according to the LDL-C control objectives in force in 2009 (<100 mg/dL). The results obtained were interpreted taking these objectives into account. The current European guideline [5] recommends a therapeutic regimen that achieves ≥50% LDL-C reduction from baseline and an LDL-C goal of <1.4 mmol/L (<55 mg/dL) for the secondary prevention of patients at very high risk of acute coronary syndromes. We highlight that if we apply the recent targets of the European guideline, which establishes a lower level of LDL-C control, the percentage of good control could be worse than the observed in this study. The American guideline [6] recommends, in general, a reduction of ≥50% from baseline LDL-C and an LDL-C goal of <70 mg/dL (1.8 mmol/L) for atherosclerotic cardiovascular disease patients. However, nowadays there are still some countries such as Japan and Macao that consider a cLDL <100 mg/dL as a good control value in secondary prevention of CVD [28,29].

Some studies have addressed the implementation of strategies based on audit and feedback. In primary care, a clinical trial was carried out with GPs that aimed to study the effectiveness of audit and feedback at improving the care of patients with chronic diseases, including IHD. No differences were found in cholesterol levels, or between GPs receiving feedback about their usual patient care in comparison with those from the intervention arm, who received feedback about patient care jointly with a goal setting and action plan [30].

A 2012 Cochrane review of the effects of audit and feedback on professional behavior and patient outcomes noted that these can range from a null to a substantial effect, and that feedback turns out to be more effective when delivered more intensively and accompanied by an action plan with well-defined objectives [31].

In our case, the face-to-face feedback session consisted of a 1 h-long oral presentation of general results from all participants, in which physicians’ individual data were not mentioned and no individualized action plans were presented. The Cochrane review reported that the effects of auditing and feedback may be greater when health professionals actively participate and have specific responsibilities for implementing change [31,32]. Unlike the face-to-face feedback session, the physicians had a more active role in the online course, because they were dealing with their own patients and had to design an action plan for improvement. The online course consisted of a theoretical part of reading the CPG, a second part about hypothetical clinical case resolution, and a final part of self-audit of patients with IHD and poor control of LDL-C in which physicians had to propose a strategy for improvement. Thus, we believe that the self-audit carried out in the online course in Phase 1 was probably responsible for the improvement in control of LDL-C, and could explain the good global results achieved in this phase. Likewise, the greater involvement and active participation of physicians, required for the success of Phase 1, could have influenced these results. Conversely, the feedback session (Phase 2) was more passive, which probably explains the worse result, at least in part. A systematic review of interventions for improving adherence to CPGs for CVD showed that more actively educational interventions can produce such statistically significant improvements and thereby be more effective than passive diffusion strategies [33].

Analyzing the factors associated with LDL-C control after Phase 1 reveals that being older and being female are the patient characteristics associated with poor LDL-C control. Some authors have reported that, overall, women with IHD receive fewer medication, specifically lipid-lowering drugs, than men do [34,35], making it difficult to achieve LDL-C control objectives. It is known that aspects such as preferences for therapeutic strategies and adherence to lifestyle and pharmacological interventions can differ by sex in a variety of health problems [36,37]. Physicians’ awareness of their patients’ risk can also be influenced by sex [38]. However, most of the available medical guidelines are not gender-specific or sex-specific and more evidence-based data is currently need to update recommendations and implement specific programs [37,39].

Conversely, in our study, being diabetic was associated with a greater probability of controlling LDL-C well. This finding suggests that diabetic patients follow more rigorous controls, provided by their GPs. Moreover, the recommendations of CPGs concerning LDL-C control in these patients are also stricter than they are for other patients [40,41].

We also found that IHD patients whose physicians are tutors in a TCGPR are more likely to have good LDL-C control after Phase 1, the probability being even higher when the physician has also completed the online course. The CPV result also shows a physician-dependent effect accounting for 3% of the variation in LDL-C control, meaning that patient control could vary depending on the physician they are assigned.

Previous studies have shown greater compliance with CPG recommendations in the IHD management in teaching hospitals compared with non-teaching hospitals, since physicians who teach are more likely to provide evidence-based care [42,43,44]. At the primary care level, patients scored higher in health surveys about managing chronic diseases if they were assigned to teaching centers for resident physicians compared with those assigned to non-teaching centers [45].

A study performed in the primary care context that compared online training in cholesterol management with face-to-face training showed that only physicians who participated in the former were likely to change their behavior. This training was associated with a significant increase in the percentage of patients with high cardiovascular risk who were treated with recommended drugs [16].

The online course undertaken in our study comprised activities that, in a previous study, were associated with better learning outcomes for health professionals [15]: carrying out practical exercises with fictional cases, interacting with teachers, self-audit and repeating practical exercises with physicians’ own real cases. Thus, the option for activities already supported by the literature may have contributed the better LDL control achieved by our study population.

Regarding the factors associated with LDL-C control after Phase 2, having had a stroke increases the probability of good control. The results from Phase 1, where being diabetic was found to favor LDL-C control, suggest that, when faced with patients with CVD or with diseases that increase cardiovascular risk, physicians probably intensify their efforts to get their patients to control their CVRFs, in this case, their LDL-C.

In 2009, only 42.8% of patients with IHD or stroke under the care of the ICS (Terres de l’Ebre territory) had good LDL-C control (data not published). Due to this unsatisfactory data and the known importance of a good secondary prevention in IHD we decided to design a quasi-experimental study and promote a training course that would reach as many GPs as possible with the aim of improving their patients’ LDL-C control. This aim justifies not using a clinical trial design.

In our study, most of the data were extracted from the ECAP computerized registry. This imposes a limitation on the study because not all patients have complete data. We excluded 52.0% and 41.1% of the patients from the 2009 and 2011 periods, respectively, because they did not have an LDL-C result for that year. These values are somewhat higher than those reported elsewhere in the literature (25.4–42.1%) [46,47]. Nevertheless, the sample size achieved was sufficiently able to evaluate the study results.

This study provides valuable information for health managers about the impact of different activities in a strategy of CPG implementation in the primary care setting. To ensure adequate adherence to CPGs in clinical practice, health systems must opt to follow evidence-based strategies if they are to facilitate better health results. Further efforts are needed to overcome barriers to the implementation of CPGs. These must consider the different contexts, intervening factors and implementation strategies with the aim of guaranteeing a positive impact on health. In addition, new studies should consider the influence of aspects of communication with patients on achieving their health objectives. These aspects include patients’ health information needs, the perception of the relevance and the degree of understanding of the health information received, and the doctor–patient communication strategies used.

## 5. Conclusions

Cholesterol control in IHD patients was influenced by the type of training activity undertaken by physicians during the implementation of the GPC, and patient and physician characteristics. Physicians working in a TCGPR promote cholesterol control among their patients, the probability of control being higher still if these GPs undertake online training with practical exercises, interactivity, self-audit, and strategic planning. Of the patients’ characteristics, being female and being older were risk factors, while being diabetic and having suffered a stroke were protective factors for LDL-C control. Of the physicians’ characteristics, being a tutor in a TCGPR was a protective factor for their patients.

## Figures and Tables

**Table 1:** Distribution of physicians’ participation in the phases of the clinical practice guidelines (CPG) implementation.

Participation by Phase	Physicians *n* (%)	Patients *n* (%)
Phase 1 (face-to-face session and online course)		
Neither activity	35 (32.4)	328 (28.5)
Face-to-face session only	29 (26.9)	325 (28.2)
Online course only	12 (11.1)	172 (14.9)
Face-to-face session and online course	32 (29.6)	326 (28.3)
Total of physicians/patients	108 (100)	1151 (100)
Phase 2 (feedback session)		
Did not attend	60 (57.1)	459 (50.3)
Attended	45 (42.9)	453 (49.7)
Total of physicians/patients	105 (100)	912 (100)

*n*: number of physicians or patients; %: percentage.

**Table 2:** Changes in low-density lipoprotein cholesterol (LDL-C) control after the development of Phases 1 and 2 of the CPG implementation.

Phase/LDL-C Control		Post-Phase 1		Post-Phase 2		*p* Value
		No *n* (%)	Yes *n* (%)	No *n* (%)	Yes *n* (%)	
Pre-Phase 1	No	363 (31.5%)	207 (18.0%)	-	-	<0.001 ^a^
	Yes	130 (11.3%)	451 (39.2%)	-	-	
Pre-Phase 2	No	-	-	149 (16.3%)	241 (26.4%)	0.128 ^a^
	Yes	-	-	174 (19.1%)	348 (38.2%)	

*n*: number of patients; %: percentage; LDL-C: low-density lipoprotein cholesterol; ^a^ McNemar’s test.

**Table 3:** Multivariate multilevel analysis showing the association between LDL-C control after Phase 1 of CPG implementation and patient and physician characteristics.

Factors (Reference Group)	OR	95% CI	*p* Value
Patient characteristics			
Sex (male)	1.59	1.22–2.08	0.001
Age	1.02	1.00–1.03	0.013
Comorbidity	0.93	0.76–1.13	0.465
Diabetes mellitus (No)	0.54	0.38–0.75	<0.001
Hypertension (No)	1.00	0.70–1.45	0.976
Stroke (No)	0.83	0.38–1.83	0.649
Peripheral artery disease (No)	1.87	0.75–4.68	0.182
Atrial fibrillation (No)	1.25	0.82–1.90	0.297
Smoker (No)	1.29	0.80–2.09	0.292
Physician characteristics			
Sex (male)	1.03	0.76–1.40	0.832
Age	1.01	0.99–1.02	0.499
Type of contract (permanent)			
Secondment	1.01	0.55–1.85	0.982
Temporary	0.86	0.49–1.52	0.604
Interim	1.02	0.73–1.43	0.913
Tutor in TCGPR (No)	0.68	0.47–0.97	0.033
Participation at Phase 1 (neither activity)			
Face-to-face only	1.04	0.74–1.45	0.827
Online course only	0.88	0.58–1.33	0.545
Both activities	1.00	0.71–1.42	0.999
Proportion of variance explained (PVE)	21.46%		
Variance partition coefficient (VPC)	2.69		
Median odds ratio (MOR)	1.33		

OR: adjusted odds ratio for all model variables; CI: 95% confidence interval.

**Table 4:** Multivariate multilevel analysis showing the association between LDL-C control after Phase 1 of CPG implementation, and the patient and physician characteristics, with the new composite variable.

Factors (Reference Group)	OR	95% CI	*p* Value
Patient characteristics			
Sex (male)	1.60	1.23–2.09	<0.001
Age	1.02	1.00–1.03	0.013
Comorbidity	0.94	0.77–1.15	0.541
Diabetes mellitus (No)	0.53	0.38–0.74	<0.001
Hypertension (No)	1.01	0.70–1.44	0.995
Stroke (No)	0.83	0.38–1.82	0.645
Peripheral artery disease (No)	1.78	0.71–4.48	0.219
Atrial fibrillation (No)	1.24	0.81–1.88	0.322
Smoker (No)	1.26	0.78–2.04	0.343
Physician characteristics			
Sex (male)	1.02	0.75–1.40	0.889
Age	1.00	0.99–1.02	0.706
Type of contract (permanent)			
Secondment	0.93	0.50–1.73	0.808
Temporary	0.90	0.50–1.62	0.723
Interim	0.95	0.67–1.34	0.770
Level of participation by TCGPR tutoring status (Not tutor, no activity)			
Not tutor, face-to-face session	0.95	0.67–1.36	0.795
Not tutor, online course	1.07	0.66–1.71	0.794
Not tutor, both activities	1.03	0.71–1.49	0.875
Tutor, no activity	0.69	0.21–2.30	0.544
Tutor, face-to-face session	0.98	0.56–1.70	0.939
Tutor, online course	0.42	0.22–0.80	0.009
Tutor, both activities	0.61	0.32–1.16	0.134
Proportion of variance explained (PVE)	21.46%		
Variance partition coefficient (VPC)	2.69		
Median odds ratio (MOR)	1.33		

OR: adjusted odds ratio for all model variables; CI: 95% confidence interval.

**Table 5:** Multivariate multilevel analysis showing the association between LDL-C control after Phase 2 of CPG implementation and patient and doctor characteristics.

Factors (Reference Group)	OR	95% CI	*p* Value
Patient characteristics			
Sex (male)	1.03	0.76–1.40	0.819
Age	0.99	0.98–1.01	0.806
Comorbidity	0.97	0.77–1.22	0.798
Diabetes mellitus (No)	0.84	0.57–1.22	0.387
Hypertension (No)	1.51	0.99–2.30	0.051
Stroke (No)	0.28	0.08–0.97	0.045
Peripheral artery disease (No)	2.00	0.70–5.75	0.191
Atrial fibrillation (No)	1.08	0.64–1.74	0.745
Smoker (No)	0.98	0.56–1.71	0.982
Physician characteristics			
Sex (male)	0.96	0.68–1.35	0.829
Age	1.01	0.99–1.03	0.289
Type of contract (permanent)			
Secondment	1.18	0.59–2.38	0.643
Temporary	1.22	0.63–2.38	0.544
Interim	1.25	0.85–1.83	0.253
Tutor in TCGPR (No)	0.84	0.64–1.13	0.261
Attendance at 2011 feedback session (No)	1.16	0.78–1.71	0.467
Proportion of variance explained (PVE)	16.92%		
Variance partition coefficient (VPC)	3.04		
Median odds ratio (MOR)	1.36		

OR: Adjusted odds ratio for all model variables; CI: 95% confidence interval.
